# Identification and Functions of JHE 6 Specifically Expressed in *Bombyx mori* Silk Gland

**DOI:** 10.3390/insects14120908

**Published:** 2023-11-27

**Authors:** Xia Zhang, Jikailang Zhang, Keli Wu, Hongguo Yang, Tingcai Cheng, Chun Liu

**Affiliations:** 1 State Key Laboratory of Resource Insects, Southwest University, Chongqing 400715, China; zxzxzx919191@163.com (X.Z.); zhang598214762@163.com (J.Z.); wkl26622662@163.com (K.W.); yanghg2023@163.com (H.Y.); chengtc@swu.edu.cn (T.C.); 2 Jinfeng Laboratory, Chongqing 401329, China

**Keywords:** juvenile hormone esterase 6, juvenile hormone, silk protein, *Bombyx mori*

## Abstract

**Simple Summary:**

The regulation of insect growth and development is intricately governed by the levels of juvenile hormone and 20-hydroxyecdysone. Juvenile hormone esterase is a key catabolic enzyme of juvenile hormone that is involved in the growth, development, and metamorphosis of insects. In this study, we identified the juvenile hormone esterase genes *Bmjhe1–9* in silkworm and found that *Bmjhe6* was specifically expressed in the silk gland. In order to explore the function of *Bmjhe6*, the expression of *Bmjhe6* was down-regulated in an RNA interference experiment. The results showed that the development of the silk gland was changed as well as the juvenile hormone pathway genes. Interestingly, *Bmjhe6* was secreted into the silk gland lumen and cocoon silk, which may have new functions. This study provides some theoretical basis for the regulation of tissue growth and development as well as silk protein synthesis by juvenile hormone esterase, and servers as a reference for the study of juvenile hormone esterase in other insects.

**Abstract:**

Juvenile hormone esterase (JHE) is the specific enzyme that degrades juvenile hormone (JH) and regulates the JH titer in insects. JH also regulates the development of the silk gland and the synthesis and secretion of silk proteins in *Bombyx mori*. Here, we identified nine possible JHE family members, Bmjhe1–9. Notably, *Bmjhe6* is specifically expressed in the silk gland. Using semi-quantitative, quantitative real-time RT-PCR and Western blot, it was confirmed that *Bmjhe6* was specifically expressed in the middle silk gland (MSG) with high levels in the anterior region of the MSG (A-MSG). The immunofluorescence localization analysis revealed that Bmjhe6 is produced within cells, secreted into the gland lumen, and co-transported with silk proteins into the anterior silk gland (ASG). In vitro hormone induction experiments demonstrated that *Bmjhe6* responds to a JH analog, increasing its expression after 12–24 h, whereas 20-hydroxyecdysone inhibited it. In addition, Bmjhe6 knockdown using dsBmjhe6 injections accelerated larval development, resulting in increased larval body and silk gland weight. This induced disordered sericin genes (*Ser2*, *Ser3*) expression, and key genes in the JH synthesis pathway (*BmKr-h1* and *BmMet1*) were significantly upregulated along with the transcription factors (*SGF-1* and *Sage*). These results indicate that Bmjhe6 plays an important role in silk gland growth and silk protein synthesis by modulating JH signal.

## 1. Introduction

Insect hormones are secreted by glands in the insect body, and hormone imbalance leads to abnormal development or even death in insects. Of these hormones, juvenile hormone (JH) and 20-hydroxyecdysone (20E) are directly involved in the development processes of insects. Under the influence of JH and 20E, insect larvae complete each molting process and maintain their morphology [1]. JH is secreted by a pair of corpora allata on the posterior side of the brain and regulates insect growth, development, metamorphosis, and reproduction [2]. The concentration of JH in the hemolymph and intracellular tissues is maintained and balanced by the synthesis and metabolism of JH. JH metabolism is mainly catalyzed by juvenile hormone esterase (JHE), juvenile hormone epoxide hydrolase (JHEH), and juvenile hormone diol kinase (JHDK) [3]. JHE, the most prominent of these enzymes, is mainly present in the hemolymph of insects and the cytoplasm of tissues (e.g., the fat body). It converts JH into juvenile hormone acid (JHa). JHEH is found in the cytoplasm of some tissues and degrades JH into juvenile hormone diol (JHd) and JHa to hormone acid diol (JHad), while JHE also degrades JHd into JHad. JHDK is present in the cytoplasm of some insects and participates in the degradation of JHd [4]. General esterases can degrade free JH but cannot degrade JH bound to hemolymph JH-binding protein (hJHBP). JHE exhibits high specificity towards JH and is capable of degrading free JH similar to general esterases, as well as JH bound to hJHBP. Furthermore, JHE recognizes the JH-JHBP complex and decomposes bound JH, facilitating its rapid removal from blood and tissues [5]. Because JH is exclusive to insects, genes or enzymes associated with JH synthesis, transport, and metabolism serve as optimal targets for biological pest control.

JHE plays a very crucial role in controlling JH titers in insects. The promotion or inhibition of JHE activity during targeted stages can affect JH levels and lead to abnormal development and growth. Xu et al. employed RNAi to decrease JHE expression in adult red flour beetles (*Tribolium castaneum*), resulting in adult atrophy and a reduced plumage rate [6]. The inhibition of JHE activity by trifluoroacetone sulfide can reduce the degradation rate of JH and delay metamorphosis in tobacco hornworm (*Manduca sexta*) [7]. JHEs have been widely investigated in lepidopteran insects, including the tobacco hornworm (*Manduca sexta*) [8], tobacco budworm (*Heliothis virescens*) [9], beet armyworm (*Spodoptera exigua*) [10], and diamondback moth (*Plutella xylostella*) [11], among other species. Among previous studies on the lepidopteran insect silkworm (*Bombyx mori*), Takahiro et al. conducted the first identification and affinity purification of silkworm JHE in 2000. Additionally, substrate specificity and inhibitor sensitivity studies have been performed on the hemolymph JHE [12]. Hirai et al. successfully cloned the silkworm JHE gene and found that the recombinant JHE protein expressed in baculoviruses was hydrolyzed 3H-JH III and a JH analogue [13,14]. It has been reported that knocking out JHE in the fat body of silkworm at the larval stage prolongs silkworm larval growth and development [15]. Moreover, JHE overexpression in transgenic silkworm strains resulted in pupal metamorphosis after the third instar [16].

Silkworm silk gland is the only organ that can synthesize and secrete silk proteins. Silk gland can be divided into the anterior silk gland (ASG), the middle silk gland (MSG) and the posterior silk gland (PSG) based on morphology and function [17]. The ASG functions solely as a channel for transporting silk proteins and does not participate in silk secretion. The MSG is the largest segment in the silk gland, and secretes sericin protein. The PSG is slender and curved and mainly synthesizes silk fibroin [18]. Silk proteins from silkworms mainly consist of sericin and fibroin. Sericin is a natural macromolecular protein that is mainly composed of Ser1, Ser2, and Ser3 proteins. Ser1 is primarily secreted in the middle region of the middle silk gland (M-MSG) and the posterior region of the middle silk gland (P-MSG) cells, Ser2 is secreted mainly in the anterior region of the middle silk gland (A-MSG) cells, and Ser3 is also secreted mainly in A-MSG cells [19]. Silk fibroin is mainly composed of silk fibroin heavy chain, light chain, and P25 protein. Silk fibroin secreted by the PSG is secreted into the gland lumen and transported to the MSG. In this region, Silk fibroin is encapsulate by sericin before being secreted out of the body via dehydration and external forces, resulting in silk formation [20,21].

The development of silk glands, along with the synthesis and secretion of silk proteins, is synergistically regulated by JH and 20E. This regulation is closely related to the concentration of JH and 20E hormones [22,23]. Additionally, Hamada et al. found that JH titers regulated the sericin gene mRNA synthesis [24]. Zhao et al. treated fifth-instar silkworm larvae with different JH III concentrations and observed an inhibitory effects on sericin gene expression [25]. Tripoulas et al. found that by injecting low concentrations of 20E into fifth-instar silkworms, the mRNA levels of sericin and fibroin genes decreased simultaneously, with the changes being more pronounced for the sericin gene [26]. These findings demonstrated that JH regulates silk protein synthesis in silkworms.

In our preliminary work, it was discovered for the first time that one of JHE was specific highly expressed in silk gland with an updated version of the silkworm genome database. To identify the JHE and explore its function in the silk gland, we conducted a preliminary analysis on and identified the JHE coding gene in the silkworm genome with SilkDB3.0 and found that BmJHE6 was specifically expressed in the silk gland. We also studied its expression, localization, and function in silk gland.

## 2. Material and Method

### 2.1. Experimental Insects

The silkworm strain Dazao was provided by the Silkworm Gene Bank (Southwest University, Chongqing, China). The silkworm larvae were fed fresh mulberry leaves and reared in incubators at a temperature of 25 °C with a relative humidity of 75% under a 12 h light and dark cycle.

### 2.2. Bioinformatic Analysis

The amino acid sequences of the unique structural domains of the typical JHE proteins (PF00135 and PF07859 were retrieved from the PFAM database. SilkDB3.0 (https://silkdb.bioinfotoolkits.net, accessed on 8 January 2020) and NCBI BLAST were used for whole-genome alignment to obtain the preselected Bmjhe genes. NCBI BatchCDD and SMART were used to compare the preselected JHE protein sequences to predict the conserved structural domains. The signal peptide was predicted using SignalP 4.1 Server4. The basic local alignment search tool and MEGA software (version 5.0) were used to perform homology analysis of the sequence and to construct the phylogenetic tree for *Bombyx mori*, *Bicyclus anynana*, *Helicoverpa armige*, *Manduca sexta*, and *Spodoptera frugiperda*. Multiple sequence alignments and phylogenetic trees generation were performed using the ClustalW2 server at EMBL-EBI (www.ebi.ac.uk/Tools/msa/clustalw2, accessed on 1 July 2022) for 14 insects with high homology. A heat map was generated using TBtools [27].

### 2.3. Reverse Transcription-Polymerase Chain Reaction (RT-PCR) and Quantitative Real Time-PCR (qRT-PCR)

Total RNA was extracted from multiple silkworm larvae tissues, including the ASG, MSG (A-MSG, M-MSG, and P-MSG), and PSG using TRIzol™ reagent (Invitrogen, Carlsbad, CA, USA). Reverse transcription was performed using a PrimeScript™ RT reagent kit (Takara, Shiga, Japan). The conditions for semi-quantitative RT-PCR were as follows: 94 °C for 30 s, followed by 30 cycles of 94 °C for 10 s, 60 °C for 15 s, and 72 °C for 90 s. The final extension was performed at 72 °C for 7 min after the last cycle. Quantitative PCR was performed using SYBR^®^ Premix Ex Taq™ II (Takara, Shiga, Japan). The qPCR reaction was performed under the following conditions: 95 °C for 10 s, followed by 40 cycles of treatment at 95 °C for 5 s, and 60 °C for 31 s. The silkworm ribosomal protein L3 (BmRpl3) was used as the internal marker. All experiments were conducted in three independent replicates. The results were analyzed by 2^−ΔΔCt^. All the primer sequences are listed in Supplementary Table S1.

### 2.4. Polyclonal Antibody Preparation and Western Blotting (WB)

A polypeptide DDDPIVLIAEGRGK of Bmjhe6 was selected as the antigen, and Rabbit polyclonal anti-Bmjhe6 antibodies were made and purified by GenScript^TM^ (Najing, China). Radio-immunoprecipitation assay (RIPA) lysis buffer (Beyotime, Shanghai, China) was used to extract proteins from multiple silkworm larvae tissues, including the ASG, MSG (A-MSG, M-MSG, and P-MSG), and PSG. The supernatant of the homogenate was collected and centrifuged at 12,000× *g* for 5 min, and the protease inhibitor was added. The protein concentrations were measured using a bicinchoninic acid (BCA) protein Assay Kit (Beyotime, Shanghai, China). The proteins (40 µg per well) were separated by 12% SDS-PAGE and transferred onto a polyvinylidene difluoride (PVDF) membrane (Roche, Basel, Switzerland). The membranes were blocked with 5% non-fat dry milk overnight at 4 °C and incubated with the primary antibodies, anti-Bmjhe6, (1:2000) for 2 h at 37 °C. After washing the membrane (6 × 5 min), the membranes were incubated with goat anti-rabbit IgG secondary antibodies labeled with horseradish peroxidase (HRP) (Sigma, St. Louis, MO, USA). The protein bands were visualized with SuperSignal™ West Femto Maximum Sensitivity Substrate (Thermo Fisher Scientific, Waltham, MA, USA) using the automatic exposing pattern of Genome XRQ (Gene Company, Hong Kong, China).

### 2.5. Immunohistochemistry

The ASG and MSG (A-MSG, M-MSG, and P-MSG) from Day-3 fifth-instar larvae were fixed in 4% paraformaldehyde for 2 h at 4 °C. The samples were embedded in paraffin and 5 µm thick sections were prepared for immunofluorescence analyses. The sections were treated with the primary anti-Bmjhe6 antibody (1:200), followed by treatment with the CY3-labeled secondary goat anti-rabbit IgG (Sigma) for anti-Bmjhe6. After washing (3 × 5 min), sections were stained with DAPI and observed under a confocal microscope (OLYMPUS FV100, Tokyo, Japan).

### 2.6. JHA and 20E Treatment

The JHA (Sangon, Shanghai, China) was dissolved in DMSO (5 μg/μL) [28]. Following this, 1 µL JHA solution was measured using a pipette and applied evenly to the backs of the Day-1 fifth-instar larvae. 1 µL DMSO was applied to the backs of Day-1 fifth-instar larvae for the control. In addition, the 20E (Oxoid, Basingstoke, UK) was dissolved in 5% ethanol (5 μg/μL). 5 µL of the solution was injected into the hemolymph through the second foot of the Day-1 fifth-instar silkworms, and an equal volume of ethanol was injected into the control larvae. Silk glands were harvested from the larvae 12 or 24 h after application for RNA extraction.

### 2.7. dsRNA Treatment

The dsRNA primers for the Bmjhe6 and EGFP genes were designed using Primer Premier (version 5.0), and the T7 promoter sequence was added to the forward and reverse primers. The primer sequences are shown in Supplementary Table S1. cDNA from the silk gland of Day-3 fifth-instar silkworms was used as a template for PCR amplification. The PCR products were used as templates for dsRNA synthesis using the T7 RiboMAX™ Express RNAi System (Promega, Madison, WI, USA). The final concentration used for RNAi-mediated knockdown was approximately 3000 ng/μL dsRNA. Day-2 second-instar, Day-1 third-instar, and Day-1 fourth-instar larvae were injected with 2 μL, 3 μL, and 5 μL Bmjhe6 dsRNA, respectively. The control group was injected with EGFP dsRNA under similar experimental conditions.

### 2.8. Statistical Analysis

All data results were expressed as mean ± standard deviations. One-way analysis of variance was performed following an unpaired two-tailed Student’s *t*-test. * *p*-value < 0.05, ** *p*-value < 0.01, and *** *p*-value < 0.001 indicated statistically significant difference.

## 3. Results

### 3.1. Bioinformatic Analysis of the JHE Family Members in the Bombyx mori Genome

**Figure 1:**
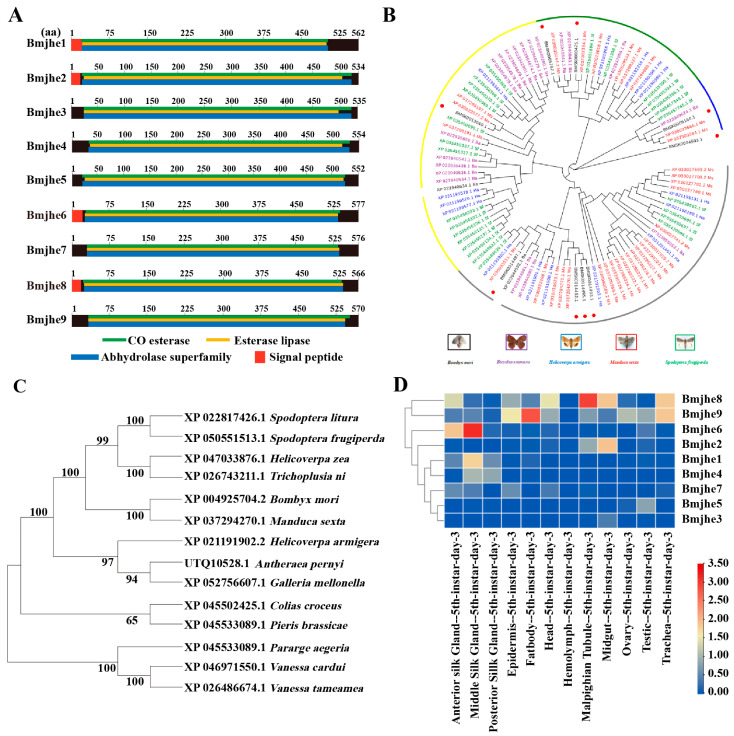
Identification of Bmjhe candidate family members and discovery of Bmjhe6. (**A**) Gene structure diagram of Bmjhe candidate family members. (**B**) Evolutionary analysis of the Bmjhe superfamily. (**C**) Phylogenetic analysis of Bmjhe proteins from 14 insects. (**D**) Microarray expression data for Bmjhe genes in various tissues of Day-3 fifth-instar silkworms.

In our preliminary work, it was found that one of JHE was special highly expressed in silk gland with an updated version of the silkworm genome database SilkDB3.0. To gain a more comprehensive understanding of JHE in silkworms, we conducted bioinformatics analysis of the JHE-coding gene. Nine potential JHE-encoding genes were found in the silkworm genome and designated *Bmjhe1*–*9*. The *Bmjhe* family encodes amino acids with lengths ranging from 534 to 577 that are abundant in acidic amino acids. All members possess CO-esterase, esterase lipase, and abhydrolase superfamily structural domains, and certain members comprise a signal peptide (Figure 1A). The *Bmjhe* genes are distributed on chromosomes 8, 10, 11, 15, 23, and 25, and are mainly clustered in strings on chromosome 25 (Figure S1A). To further analyze the evolutionary relationships of the gene families, *Bombyx mori* (*Bm*), *Bicyclus anynana* (*Ba*), *Helicoverpa armiger* (*Ha*), *Manduca sexta* (*Ms*), *and Spodoptera frugiperda* (*Sf*) were identified, as well as 9 Bmjhe. Among the 14 insects with high homology, the silkworm JHE was evolutionarily most closely related to the *Manduca sexta* JHE (Figure 1B,C).

To investigate the expression pattern of *Bmjhe* family genes in various tissues in Day-3 fifth-instar larvae, we searched the SilkDB3.0 database and found that *Bmjhe* genes were mainly expressed in the MSG, fat body, and the Malpighian tubule, with *Bmjhe6* being specifically expressed in the silk gland (Figure 1D). Analysis of the specific expression period in the MSG revealed elevated gene expression during the instar stage, which declined during the molting stage and at the end of the fifth instar (Figure S1B). The full-length *Bmjhe6* cDNA was 1899 bp. The 1734 bp open reading frame encoded 577 amino acid residues and a signal peptide (Figure 1A). Protein subcellular localization prediction was performed using SilkDB3.0 and PSORT II Prediction, suggesting that Bmjhe6 is a secretory protein that is synthesized in all parts of the cell and is highly concentrated in the endoplasmic reticulum and extracellular regions (Figure S1C).

### 3.2. Bmjhe6 Expression Patterns and Localization

**Figure 2:**
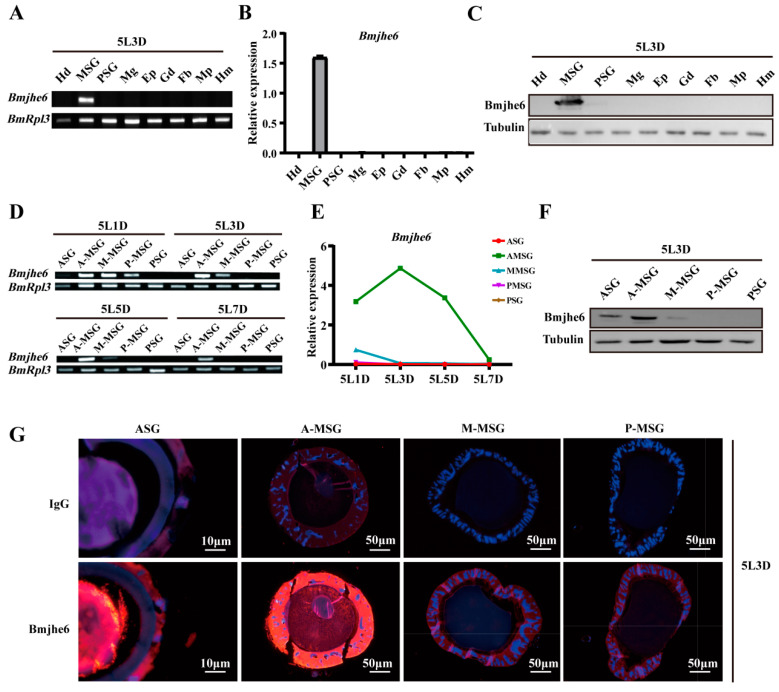
Expression and localization of Bmjhe6. (**A**) The mRNA expression of *Bmjhe6* in the various tissues of Day-3 fifth-instar silkworms using RT-PCR, (**B**) qRT-PCR, and (**C**) WB. (**D**) The mRNA expression of *Bmjhe6* in different regions silk glands of fifth-instar silkworms using RT-PCR and (**E**) qRT-PCR. (**F**) The protein expression of *Bmjhe6* in tissues of Day-3 fifth-instar silkworms using WB. (**G**) Protein localization of Bmjhe6 in different regions of the silk gland of Day-3 fifth-instar silkworms. Hd: Head; Fb: fat body; Mg: midgut; Ep: epidermis; Gd: gonad; Mp: Malpighian tubule; Hm: Hemolymph; 5L1-7D: Day-1–7 fifth instar.

To further confirm the *Bmjhe6* expression, RT-PCR, qRT-PCR, and WB were performed. The results showed that *Bmjhe6* was specifically expressed in the silk gland of Day-3 fifth-instar larvae and highly expressed in the MSG (Figure 2A–C). Almost no expression was observed in the ASG and PSG (Figure S2A,B). Among the different MSG divisions, *Bmjhe6* expression was highest in the A-MSG and lower in the M-MSG and P-MSG (Figure 2D–F). Over time, *Bmjhe6* was expressed during the instar stage and decreased during the molt stage in fourth and fifth instars, which was consistent with the expression pattern of the silk protein (Figure 2D–F and Figure S2A,B). Immunofluorescence results showed a strong fluorescent signal in the cell layer and lumen of the A-MSG, a weaker signal in the M-MSG and P-MSG, and a strong signal in the ASG lumen (Figure 2G), suggesting that Bmjhe6 was secreted along with silk fiber. Hence, the presence of this protein was evident in the cocoon proteomic data (Table S2), indicating that Bmjhe6 is transported into the silk gland lumen as silk proteins are secreted.

### 3.3. The Response of Bmjhe6 to JH and 20E

**Figure 3:**
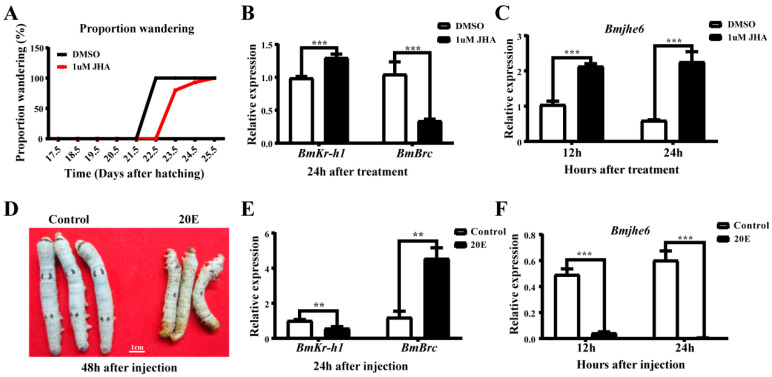
Effects of JHA and 20E on growth of silkworm and *Bmjhe6* expression of silk gland. (**A**) Larval wandering stage after JHA treatment. (**B**) Expression of *Bmkr-h1* and *BmBrc* at 24 h after JHA treatment. (**C**) Expression of *Bmjhe6* at 12 h and 24 h after JHA treatment. (**D**) Observation of larval morphology 24 h after 20E treatment. (**E**) Expression of *Bmkr-h1* and *BmBrc* at 24 h after 20E treatment. (**F**) Expression of *Bmjhe6* at 12 h and 24 h after 20E treatment. Results are expressed as means ± S.D. of three independent experiments. ** *p* < 0.01; *** *p* < 0.001.

According to the temporal expression profile of *Bmjhe6*, its expression decreased during the molting stage and increased during the instar stage, which is similar to the expression pattern of silk proteins and associated with influence of JH and 20E during the molting-instar period of the silkworm [18]. Accordingly, to investigate the response of this gene to the hormones, the silkworms were treated with JHA and 20E. After application of JHA on the backs of Day-1 fifth-instar larvae, the wandering stage was delayed by 1–2 days in the JHA group compared to that in the DMSO group (Figure 3A). Further, 24 h after JHA application, the expression of *Bmkr-h1*, a key gene in the JH pathway, was significantly increased in the JHA group, and the expression of *BmBrc*, a 20E response factor, was decreased (Figure 3B). The *Bmjhe6* expression in the silk gland was significantly increased 12 and 24 h after JHA application (Figure 3C), indicating that the expression of *Bmjhe6* in the silk gland was promoted by JHA. Similarly, 20E solution was injected into Day-1 fifth-instar larvae, and 48 h after injection, the larvae in the 20E group died because they could not molt successfully (Figure 3D). *BmBrc* expression was significantly increased in the 20E group, while *Bmkr-h1* expression was decreased 24 h after injection (Figure 3E). In the 20E group, *Bmjhe6* expression was consistently decreased from 12 to 24 h after 20E injection (Figure 3F), suggesting that *Bmjhe6* expression was inhibited by 20E. These results suggest that the *Bmjhe6* expression is stimulated by JH and inhibited by 20E.

### 3.4. The Effects on Silk Gland and the Expression of Silk Protein Genes after JHE Knockdown

**Figure 4:**
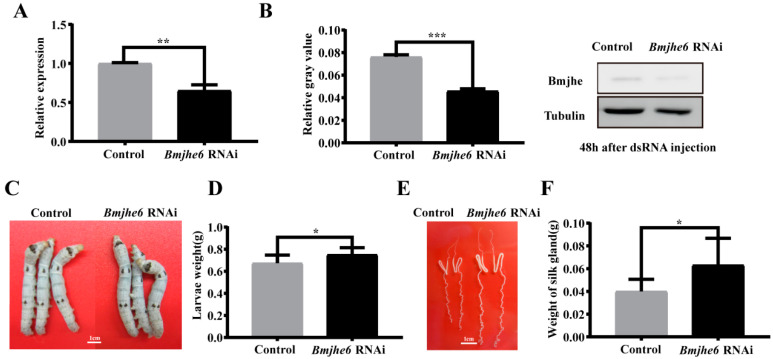
Effect of RNAi *Bmjhe6* on the growth of silkworm and silk gland. (**A**) The mRNA expression and (**B**) protein expression of *Bmjhe6* after RNAi 48 h. (**C**) Larvae size and (**D**) weight in Day-3 fifth-instar *Bmjhe6* RNAi silkworms. (**E**) Silk gland size and (**F**) weight in Day-3 fifth-instar *Bmjhe6* RNAi silkworms. Results are expressed as means ± S.D. of three independent experiments. * *p* < 0.05; ** *p* < 0.01; *** *p* < 0.001.

To further investigate the role of *Bmjhe6* in the silk gland, we examined its effect on silk gland development and silk protein synthesis using RNAi experiments during the larval stage. Through the continuous injection of *Bmjhe6* dsRNA in the larval stage, it was found that the transcription and translation levels of Bmjhe6 were significantly reduced in RNAi group during the fourth instar (Figure 4A,B), indicating that the interference of Bmjhe6 was successful. When the silkworms were reared to the Day-3 fifth instar, the larvae of the *Bmjhe6* RNAi group were larger and heavier than those of the control group (Figure 4C,D). Additionally, the *Bmjhe6* RNAi group exhibited a larger and longer silk gland morphology that was significantly heavier than that of the control group (Figure 4E,F). In addition, the survival and phenotypic rates of *Bmjhe6* RNAi were determined (Table 1), and the results showed that the survival rate of *Bmjhe6* RNAi was 87%, and that the phenotypic rate was 100%, whereas the survival rate of the control group was 93%, and the phenotypic rate was 0%. These results imply that Bmjhe6 affects the growth and development of silk glands, specifically that knocking down *Bmjhe6* promotes silk gland growth and development.

**Figure 5:**
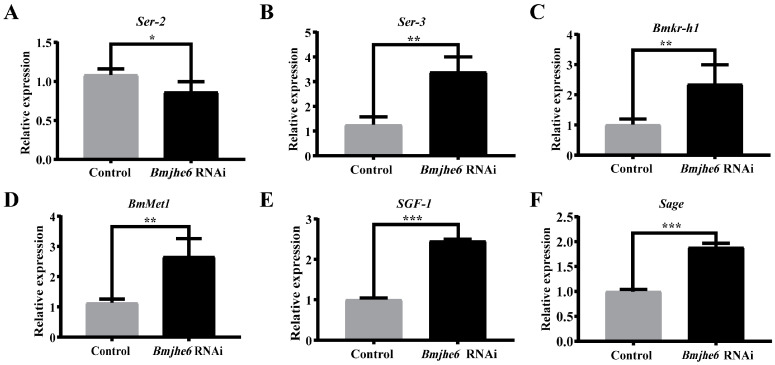
The mRNA expression of related genes in the *Bmjhe6* RNAi in A-MSG, including sericin genes (**A**) *Ser2*, (**B**) *Ser3*, key genes in the JH synthesis pathway, (**C**) *BmKr-h1*, (**D**) *BmMet1,* and transcription factors (**E**) *SGF1* and (**F**) *Sage*. Results are expressed as means ± S.D. of three independent experiments. * *p* < 0.05; ** *p* < 0.01; *** *p* < 0.001.

Silk gland tissues are the main sites for the synthesis and storage of silk proteins [24]. To investigate the effect of *Bmjhe6* on silk protein synthesis, the expression of sericin genes was investigated in the A-MSG of the Day-3 fifth-instar silkworms. In the *Bmjhe6* RNAi group of silkworms, the expression of *Ser2* and *Ser1* was significantly reduced, whereas that of *Ser3* was significantly increased (Figure 5A,B and S3). Because JHE in silkworm fat bodies can degrade JH, it was hypothesized that if *Bmjhe6* also performs the same function, it should affect the JH titer in the silk gland and the expression of genes such as *Kr-h*. The results demonstrated that the early response factor *Bmkr-h1* and JH-binding receptor *BmMet1* were both significantly upregulated (Figure 5C,D). In addition, the transcription factors *SGF1* and *Sage* (Figure 5E,F), which regulate sericin genes, were also elevated. These results suggest that Bmjhe6 affects sericin synthesis by modulating JH signal, leading to the disruption of sericin gene expression.

## 4. Discussion

JHE is a key catabolic enzyme of JH that is involved in the growth, development, and metamorphosis of insects [29]. In present study, nine JHEs were identified and analyzed using the SilkDB3.0 database for silkworm (Figure 1). It was found that *Bmjhe6* was specifically expressed in the silk gland. Further results indicate that *Bmjhe6* was mainly highly expressed in the anterior part of the MSG, and that the Bmjhe6 protein was secreted into the silk gland lumen, which was secreted into the silk fiber (Figure 2). Moreover, *Bmjhe6* expression was increased by JHA and inhibited by 20E (Figure 3). At the individual level, *Bmjhe6* knockdown resulted in the increased expression of *Kr-h1* and *Met1*, which are the early response factor and receptor of the JH pathway, respectively. Additionally, the expression of sericin genes was significantly changed (Figure 5). Our results suggest that *Bmjhe6* plays an important role in the regulation of silk synthesis by regulating the JH signal pathway.

In our study, nine JHE-coding genes were identified in silkworm genome, which exhibited different tissue expression patterns during the fifth instar. For example, *Bmjhe6* was specifically expressed in the silk gland, *Bmjhe8* was mainly expressed in the Malpighian tubule, and *Bmjhe9* was expressed in the fat body (Figure 1D), suggesting that they play functional roles in these tissues. Previous studies have reported that *Bmjhe9* can degrade JH [30], however, it is still unknown whether *Bmjhe6* also has similar functions in the silk gland. Following Bmjhe6 knockdown during the larval stage, we observed that the silk glands were larger than those in the control group (Figure 4E,F). In addition, The expression of the *Kr-h* was upregulated, which is consistent with the results of individual treatments with JHA [28], indicating that JH signal in the silk gland is stronger in *Bmjhe6* RNAi individuals than in the control group. Therefore, we speculate that *Bmjhe6* likely participates in the breakdown of JH in the silk gland.

Continuous RNAi administration was used to investigate the functions of JHE. Given the uncertain effectiveness of RNAi in individual silkworms, we continuously injected dsRNA and detected the Bmjhe6 expression at RNA and protein levels. Its expression was significantly decreased, and a phenotype observed (Figure 4). Therefore, we believe that the RNAi results are reliable. Correspondingly, Cai et al. observed significant changes in the ASG of the silkworm after injecting POU-M2 dsRNA during the larval stage [31], indicating that RNAi administration was effective during the early larval stage. We believe that the effects of RNAi on the silkworm larvae are related to the functions of the gene. Some genes have complementary homologous genes or do not play important roles, therefore, even when they are knocked out, it is difficult to observe mutant phenotypes. In addition, some genes have specific expression sites, long expression periods, or high expression levels, and it is difficult to observe phenotypes after RNAi treatment, such as silk genes. Furthermore, it is difficult to observe phenomenon during intervention at the fifth-instar stage, and even at the molecular level. In our study, we continued RNAi administration during the early of larval stages and observed clear phenotypes in the early fifth-instar stage. However, this change gradually disappeared with the later growth, therefore, when the RNAi individuals were mounted, the economic properties of the cocoons were not significantly different from those of the control group. If further research is needed, it is necessary to knock out the gene, which we are presently engaged in.

It was found that Bmjh6 was secreted into the silk gland lumen and cocoon silk (Figure 2G and Table S2). As a catabolic enzyme of JH, it is difficult to understand why it is secreted into the cocoon silk. The cocoon contains many proteases and protease inhibitors that prevent silk protein from being destroyed by other organisms and protect the pupae from developing into moths. Therefore, one possibility is that Bmjh6 may be involved in the epidemic prevention mechanisms of cocoons. Bmjh6 in cocoon silk can decompose JH in insects, leading to abnormal development in insects after the insects consume cocoon silk. Because animals instinctually seek benefits and avoid harm, insects generally do not consume cocoon silk, which may be one of the reasons why it can be preserved for a relatively long time. Another possibility is that Bmjh6 is involved in the formation of cocoon silk fibrosis because Bmjh6 possesses CO esterase that can cleave esters into acids and alcohols through hydrolysis with the participation of H_2_O. During the process of neutral silk fibroin solution passing through the MSG and ASG from the PSG, it is heavily dehydrated and coagulated owing to the acidic environment of in the MSG and ASG, ultimately completing the cocoon silk fibrosis process [20,21]. Therefore, Bmjhe6 may not only decompose JH in the silk gland, but also participate in the process of cocoon silk fibrosis. However, this hypothesis warrants further verification.

Unfortunately, our study lacked direct evidence for the degradation of JH by Bmjhe6. In fact, we also obtained soluble Bmjhe6 with prokaryotic cells. It was found that the concentration of JH III decreased with the increase in JHE concentration, but the degree of this decreasing was not positively correlated with the concentration of JHE. However, the decomposition products of JH III were unable to be detected with HPLC. It could possibly be caused by the low or almost no activity of Bmjhe6. In our next work, we will study its exact relationship with JH by active Bmjhe6 using other expression systems. In addition, the JH concentration in silk gland also needs further confirmation.

In summary, our research provides insights regarding the function of *Bmjh6* and evidence for the regulation of silk gland development and silk protein synthesis by JH in silkworms. At the same time, it also provides a reference for the study of other insects’ JHE.

## Figures and Tables

**Table 1:** Statistics of deformed silk gland.

dsRNA	Number of RNAi Silkworm	Survival Rate (%)	Phenotypic Penetrance (%)
*Bmjhe6* dsRNA	30	87	100
*EGFP* dsRNA	30	93	0

## Data Availability

The data presented in the study are available in the article.

## Supplementary Materials

The following supporting information can be downloaded at: https://www.mdpi.com/article/10.3390/insects14120908/s1, Figure S1: Chromosomal localisation of *Bmjhe* candidate family members and Bmjhe6 correlation analysis; Figure S2: Expression of *Bmjhe6*; Figure S3: The mRNA expression of *Ser1* in the *Bmjhe6* RNAi in A-MSG; Table S1: Primer sequences used in this study; Table S2: Identified proteins from IV-E silk and cocoon silk.
