# Supplementary material for: Identification and Functions of JHE 6 Specifically Expressed in Bombyx mori Silk Gland
Source: Insects. 2023 Nov 27;14(12):908. doi: 10.3390/insects14120908 (PMC10743834; doi:10.3390/insects14120908)
Supplement: Supplementary file 1 [file insects-14-00908-s001.zip › Supplementary Materials.pdf]

Supplementary Materials

Table S1. Primer sequences used in this study.

Table S2. Identified proteins from IV-E silk and cocoon silk.

Figure S1. Chromosomal localisation of *Bmjhe* candidate family members and Bmjhe6 correlation analysis.

Figure S2. Expression of *Bmjhe6*.

Figure S3. The mRNA expression of *Ser1* in the *Bmjhe6* RNAi in A-MSG.

Table S1. Primer sequence

Gene	Forward primer	Reverse primer
Bmjhe6-RT-PCR	TTTTTATCCCTTGCTTCGGA	TGGGGTCGTCATCAATAATC
BmRpl3-RT-PCR	TCGTCATCGTGGTAAGGTCAA	TTTGTATCCTTTGCCCTTGGT
Bmjhe6-qPCR	ACAGATTGGCGGTTTTCGGA	CCCTTAGCGGCATCAGACAT
BmRpl3-qPCR	TTCGTACTGGCTCTTCTCGT	CAAAGTTGATAGCAATTCCCT
Ser1-qPCR	CCTCTGTAAGCAGTAAGGATAG	TTGTTTGAGTAAGCAACGGTG
Ser2-qPCR	AAGACACAGATAAGACATTCGACA	TTCAAACCACCGTTATCCTCA
Ser3-qPCR	GCCTTTGGGCGTTTACAGAC	TCTGCTCCTGAATCGAGACG
SGF1-qPCR	ATCCGACATTCGCTGTCCTT	TGACGTCGCAAGAAACAACC
Sage-qPCR	AGCAATCACGAAGGTCCGC	CGTATCGTGGTTGGAGTCGT
Kr-h1-qPCR	CTTCCTCCTACTCCACCT	GGCAACGAAATGTAATGT
Met1-qPCR	AATCTTGCCACCAACAGC	ACCCAACGCACATCTTCT
Brc-qPCR	TCGCTGACAAACACGCTG	ATGGTAAGAACGGCGGAC
ds*Bmjhe6*	TAATACGACTCACTATAGGAAGGTGGAGATTGCCGAA	TAATACGACTCACTATATTCCGGGTAACAACGGTCTG
ds*EGFP*	TAATACGACTCACTATAGGGACGACGGCAACTACAAGACC	TAATACGACTCACTATAGGGCTCGTTGGGGTCTTTGCTCA

Table S2. Identified proteins from IV-E silk and cocoon silk.

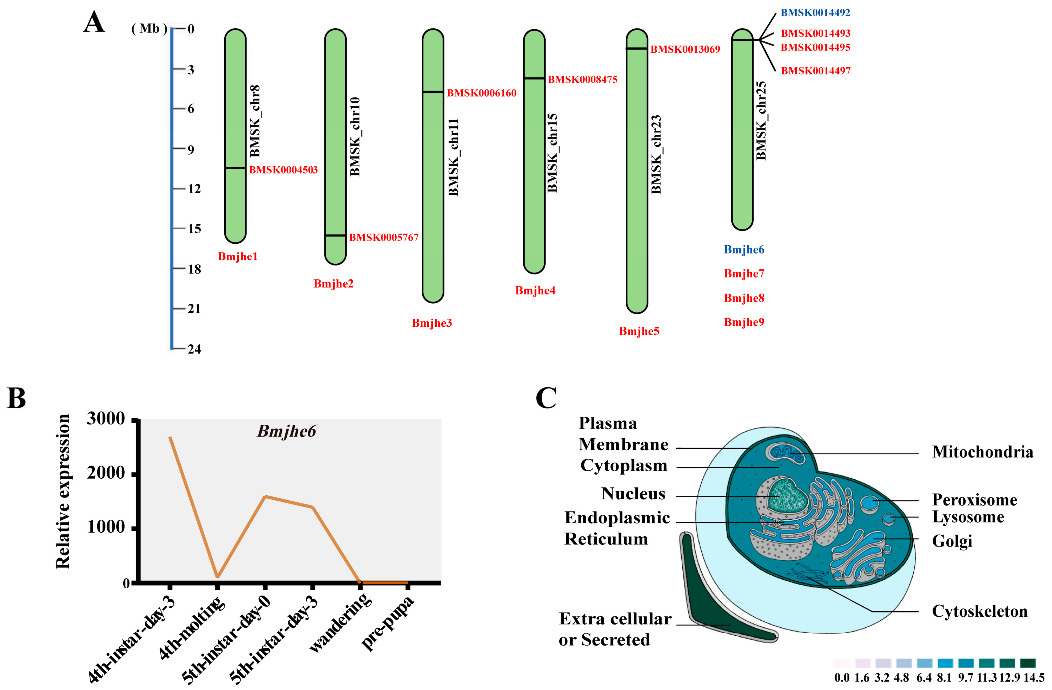

Figure S1 Chromosomal localisation of *Bmjhe* candidate family members and *Bmjhe6* correlation analysis. (A) Chromosome localization of *Bmjhe*. (B) Expression of *Bmjhe6* in the MSG. (C) Protein localization of Bmjhe6 in cells.

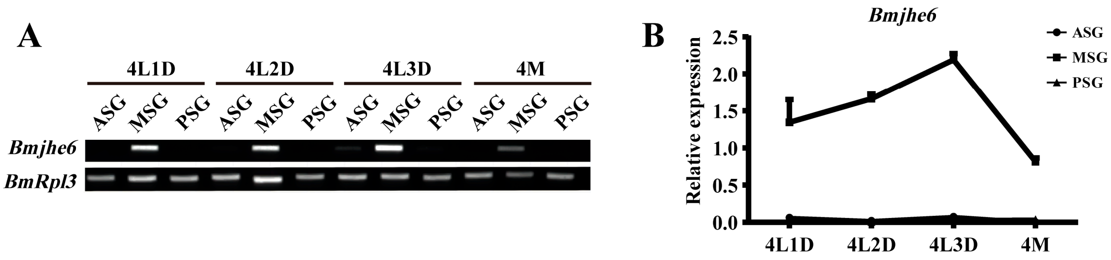

Figure S2 Expression of *Bmjhe6*. (A) The mRNA expression of *Bmjhe6* in different regions silk glands of fourth instar silkworms using RT-PCR and (B) qRT-PCR.

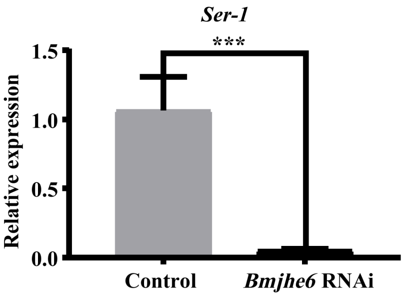

Figure 3S. The mRNA expression of *Ser1* in the *Bmjhe6* RNAi in A-MSG.
